# *Morus alba* L. suppresses the development of atopic dermatitis induced by the house dust mite in NC/Nga mice

**DOI:** 10.1186/1472-6882-14-139

**Published:** 2014-04-23

**Authors:** Hye-Sun Lim, Hyekyung Ha, Hoyoung Lee, Jun Kyung Lee, Mee-Young Lee, Hyeun-Kyoo Shin

**Affiliations:** 1 Herbal Medicine Formulation Research Group, Korea Institute of Oriental Medicine, 1672 Yuseong-daero, Yuseong-gu, Daejeon 305-811, Republic of Korea

**Keywords:** Atopic dermatitis, Keratinocyte, Macrophage, *Morus alba*, NC/Nga mouse

## Abstract

**Background:**

*Morus alba*, a medicinal plant in Asia, has been used traditionally to treat diabetes mellitus and hypoglycemia. However, the effects of *M. alba* extract (MAE) on atopic dermatitis have not been verified scientifically. We investigated the effects of MAE on atopic dermatitis through *in vitro* and *in vivo* experiments.

**Methods:**

We evaluated the effects of MAE on the production of nitric oxide (NO) and prostaglandin E_2_ (PGE_2_) in RAW 264.7, as well as thymus and activation-regulated chemokine (TARC/CCL17) in HaCaT cells. In an *in vivo* experiment, atopic dermatitis was induced by topical application of house dust mites for four weeks, and the protective effects of MAE were investigated by measuring the severity of the skin reaction on the back and ears, the plasma levels of immunoglobulin E (IgE) and histamine, and histopathological changes in the skin on the back and ears.

**Results:**

MAE suppressed the production of NO and PGE_2_ in RAW 264.7 cells, as well as TARC in HaCaT cells, in a dose-dependent manner. MAE treatment of NC/Nga mice reduced the severity of dermatitis and the plasma levels of IgE and histamine. MAE also reduced the histological manifestations of atopic dermatitis-like skin lesions such as erosion, hyperplasia of the epidermis and dermis, and inflammatory cell infiltration in the skin on the back and ears.

**Conclusion:**

Our results suggest that MAE has potent inhibitory effects on atopic dermatitis-like lesion and may be a beneficial natural resource for the treatment of atopic dermatitis.

## Background

Atopic dermatitis is a multifactorial disease that depends on a genetic predisposition to allergy, which is characterized by complex immune mechanisms in the development of allergic skin inflammation [1]. Complex interrelationships between genetic, environmental, skin barrier, pharmacological, psychological and immunological factors contribute to the pathogenesis of atopic dermatitis, but its immunological basis is of considerable importance, and it has been studied extensively [2]. Immunological analyses of atopic dermatitis have shown that the activation of mast cells and differentiated T-helper 2 (Th2) cells by various mediators have major roles in the development of dermatitis [3,4]. Previous studies have shown that atopic dermatitis is associated with increased serum immunoglobulin (Ig) E levels, the Th2 chemokine level in the lesional skin, the serum eosinophil count, and the expression of proinflammatory enzymes such as nitric oxide (NO) and prostaglandin E_2_ (PGE_2_) [5,6].

*Morus alba* L. (Moraceae) is a traditional medicinal herb used for the prevention and treatment of several diseases, such as jaundice, hematemesis, and pollakisuria [7,8]. Phytochemical studies of *M. alba* have identified alkaloids, flavonoids, glycosides, terpenoids, steroids, volatile oils and tannins [9-11]. The biological activities of *M. alba* include antidiabetic, hypolipidemic, antihypertensive, antimicrobial, antioxidant, antiatherosclerotic, anticancer, neuroprotective, and antiulcer effects [12-16]. However, there was no study on the effects of *M. alba* on atopic dermatitis. Therefore, we investigated the effect of *M. alba* extract (MAE) on NO and PGE_2_ production in RAW 264.7 macrophages, and thymus and activation-regulated chemokine (TARC) production in HaCaT keratinocytes. We examined the effects of MAE on NC/Nga mice as a model of house dust mite-induced atopic dermatitis. We measured the skin severity score, histological changes in the skin, including mast cell infiltration, and the plasma IgE and histamine levels. The effect of MAE might be associated with the suppression of the inflammatory response in atopic dermatitis.

## Methods

### Reagents and materials

Dried *M. alba* (200 g) was extracted three times with 70% ethanol (2 L) by sonication for 60 min. The extract was filtered, evaporated to dryness, and freeze-dried (11.35 g). The yield was 5.68%. The *M. alba* used in this experiment was purchased from HMAX (Jecheon, Korea) in October 2008. The origin of the sample was confirmed taxonomically by Professor Je-Hyun Lee, Dongguk University, Gyeongju, Republic of Korea. A specimen (2008-ST12) has been deposited in the Basic Herbal Medicine Research Group, Korea Institute of Oriental Medicine.

Biostir-AD®, an ointment that contains house dust mite (*Dermatophagoides farina*) extract, was purchased from Biostir Inc. (Kobe, Japan), while 0.1% Tacrolimus (Protopic®; Astellas, Grand Island, NY, USA) was used as a the positive control.

### Cell culture

The murine macrophage RAW 264.7 cells and human keratinocyte HaCaT cells were obtained from the American Type Culture Collection (Rockville, MD, USA). They were cultured in Dulbecco’s modified Eagle’s medium (Gibco Inc., Grand Island, NY, USA) supplemented with 5.5% (for Raw 264.7) or 10% (for HaCaT) heat-inactivated fetal bovine serum (Gibco Inc.), penicillin (100 U/mL), and streptomycin (100 μg/mL) in a 5% CO_2_ incubator at 37°C.

### Cell viability

Cell viability was assessed with the CCK-8 assay (Cell Counting Kit-8, Dojindo Laboratories, Kumamoto, Japan) according to the manufacturer’s instructions. RAW 264.7 cells (3 × 10^3^ cells/well) and HaCaT cells (1 × 10^3^ cells/well) were incubated in 96-well plates with various concentrations of the MAE for 24 h. CCK-8 reagent was added to each well and incubated for 4 h. The absorbance was measured at 450 nm with a Benchmark plus microplate reader (Bio-Rad Laboratories, Hercules, CA, USA). The percentage of cell viability was calculated with the following formula: cell viability (%) = (mean absorbance in test wells/mean absorbance in control wells) × 100.

### Measurement of nitrite and PGE_2_ production

RAW 264.7 cells were plated at a density of 2.5 × 10^5^ cells/well on 48-well plates and incubated overnight. The cells were treated with lipopolysaccharide (LPS, 1 μg/mL) in the absence or presence of various concentrations of MAE. After incubation for 24 h, the supernatants were used to determine the levels of nitrite (Griess Reagent System, Promega Co., Madison, WI, USA) and PGE_2_ (PGE_2_ enzyme linked immunosorbent assay (ELISA) kit, Cayman Chemical Co., Ann Arbor, MI, USA).

### Measurement of TARC production

HaCaT cells (1 × 10^6^ cell/well) were cultured on six-well plates. After reaching a confluent state, the cells were washed and treated with MAE in 1 mL of serum-free medium containing tumor necrosis factor (TNF)-α and interferon (IFN)-γ (each 10 ng/mL; R&D Systems Inc., Minneapolis, MN, USA) for 24 h. The supernatants were harvested, and TARC production was quantified using an ELISA kit (R&D Systems Inc., Minneapolis, MN, USA).

### Experimental animals

Male NC/Nga mice (eight weeks old) were purchased from Central Laboratory Animal Inc. (Seoul, Korea). The animals were housed in an air-conditioned room and maintained at 24 ± 2°C and 55 ± 15% humidity. All experimental procedures were carried out in accordance with the NIH Guidelines for the Care and Use of Laboratory Animals and were approved by Korea Institute of Oriental Medicine Institutional Animal Care and Use Committee (Approval number: #10-052).

### Induction of atopic dermatitis in NC/Nga mice

Atopic dermatitis-like skin lesions were induced in 10-week-old male NC/Nga mice using Biostir-AD, as described by the manufacturer with some modifications [17]. Briefly, the upper back was shaved, and 200 μL of 4% (w/v) sodium dodecyl sulfate (SDS) was applied to the shaved dorsal skin and both surfaces of each ear for barrier disruption. After 2 h, 50 mg of Biostir-AD was applied topically and twice per week for four weeks. The mice were allocated to four random groups (n = 7/group): normal control group (vehicle-treated mice; 70% ethanol/PBS (ratio 1:9), 200 μL/mouse), Biostir group (Biostir-AD plus vehicle-treated mice), Tacrolimus group (Biostir-AD plus Tacrolimus ointment-treated mice; 50 μg/mouse), and MAE group (Biostir-AD plus MAE-treated mice; 10 mg/mouse). The effective dose of MAE was determined according to preliminary study. We have used 10 mg/kg and 20 mg/kg of MAE as experimental doses in preliminary study. The procedure of preliminary study was same to present study. The skin severity significantly decreased in both 10 and 20 mg/kg of MAE compared with the animal with atopic dermatitis. However, the skin severity of 10 mg/kg more decreased than that of 20 mg/kg. MAE was dissolved 70% ethanol/PBS (ratio = 1:9) and applied every day for four weeks. The mice were sacrificed under anesthesia with pentobarbital (200 μg/mouse, i.p.) on the day of the experiment. During the autopsy, blood was collected from the posterior *vena cava*, and the back skin and each ear were excised for the histopathological analyses.

### Evaluation of skin severity

The relative severity of dermatitis was assessed macroscopically according to the Eczema Area and Severity Index (EASI) scoring system: 0, no symptoms; 1, mild symptoms; 2, moderate symptoms; and 3, severe symptoms. The dermatitis score was defined as the sum of the scores for erythema/hemorrhage, edema, excoriation/erosion, and scaling/dryness [18]. The range of dermatitis score is 0 to 12. The mice were also photographed once each week. The dermatitis scoring is conducted throughout blind test. Two veterinarian and three animal study experts participated in blind test.

### Histopathology

After sacrifice, the back skin and one ear of each mouse were fixed in 10% (v/v) neutral buffered formalin for 24 h at 4°C. Tissue samples were embedded in paraffin and thin-sectioned (4 μm thickness). The sections were stained with hematoxylin and eosin (H&E) solution (Sigma-Aldrich, St. Louis, MO, USA) and mounted under cover slips using Dako-mounting medium (Dako Cytomation, Glostrup, Denmark). The stained sections were photographed using a photometric Quantix digital camera, and montages were assembled using Adobe Photoshop. To measure the degree of mast cell infiltration, the skin sections were stained with toluidine blue, and the numbers of mast cells were counted in four sites. The histological alternation of tissue was examined by two histopathologists.

### Measurement of plasma IgE and histamine levels

Blood samples were drawn from the mice, separated by centrifugation at 10000 × g for 10 min at 4°C, and stored at -80°C. The levels of histamine (Oxford Biomedical Research, Oxford, MI, USA) and IgE (Bethyl Laboratories Inc., Montgomery, TX, USA) were measured in the plasma using ELISA kits, according to the manufacturer’s instructions.

### Statistical analyses

All of *in vitro* experiments were performed at least three times. The dermatitis score was analyzed using Mann-Whitney’s U test. The concentration of IgE, histamine, PGE_2_, nitrite and TARC were analyzed by one-way analysis of variance (ANOVA) followed by Dunnett’s test. The difference was considered significant at *P* < 0.05.

## Results

### MAE inhibits nitrite and PGE_2_ production in LPS-stimulated RAW264.7 cells

**Figure 1:**
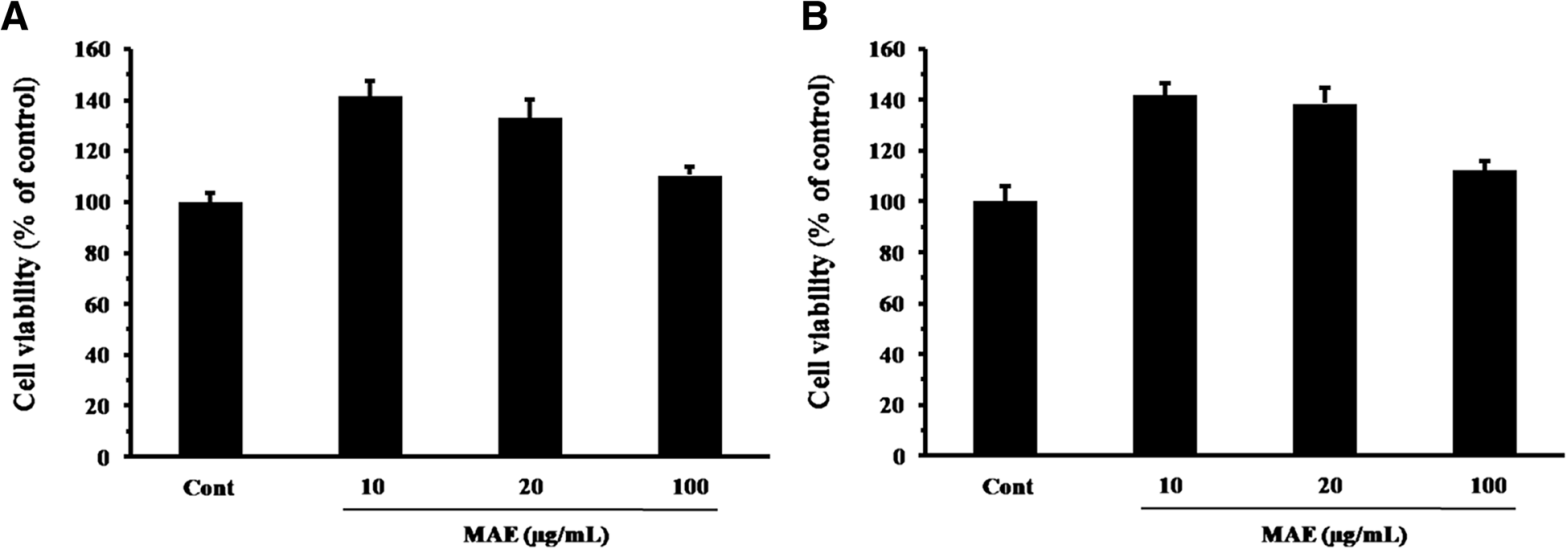
**Cytotoxicity of MAE in RAW 264.7 (A) and HaCaT cells (B).** Cells were seeded into 96-well plates and treated with MAE (10, 20, or 100 μg/mL) for 24 h. Cell viability was assessed using a CCK-8 assay.

**Figure 2:**
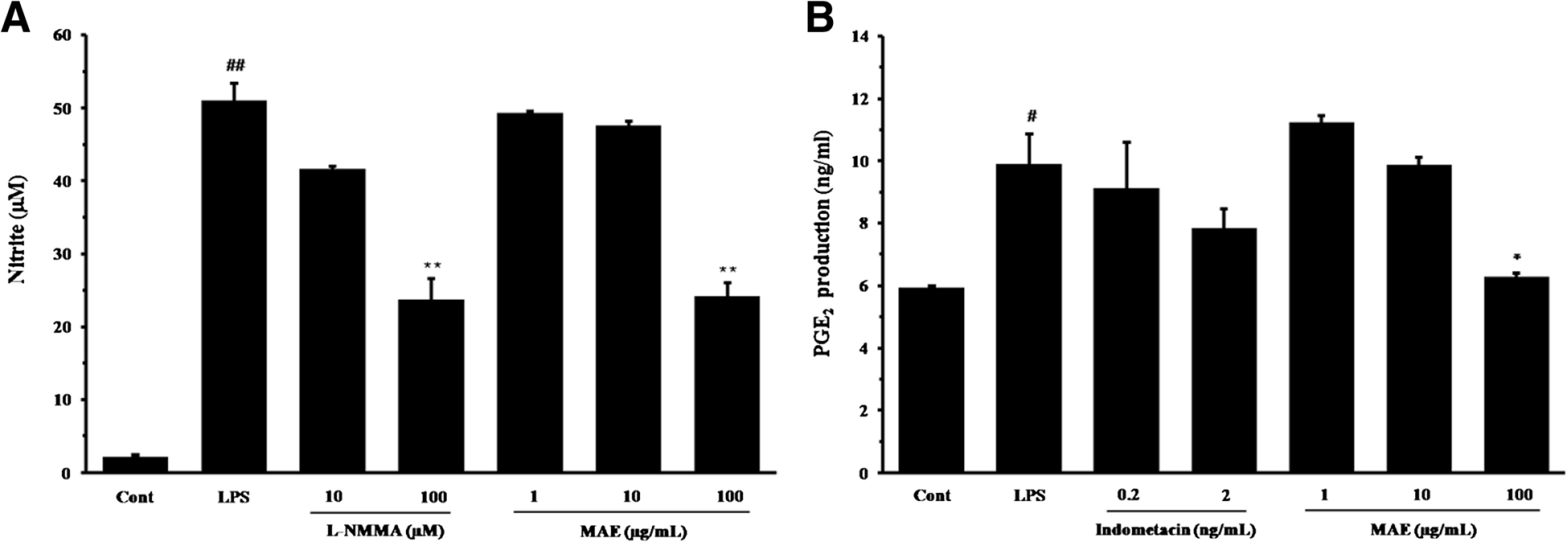
**Effects of MAE on LPS-stimulated nitrite and PGE**_**2 **_**production in RAW 264.7 cells.** The productions of nitrite **(A)** and PGE_2_**(B)** was measured using the culture supernatant from cells co-treated with MAE (1, 10, or 100 μg/mL) and LPS (1 μg/mL) for 24 h. L-NMMA (10 and 100 μM) and indomethacin (0.2 and 2 ng/mL) were used as positive control drugs. The bar groups represent means from three independent experiments. ^##^*P* < 0.01 vs. vehicle control group; ^*, **^P < 0.05 and P < 0.01 vs LPS-treated cells, respectively.

The cytotoxicity of MAE was determined in RAW 264.7 and HaCaT, subsequent experiments were performed in cells at non-toxic concentrations (Figure 1). To determine the effects of MAE on nitrite and PGE_2_ production, RAW264.7 cells were treated with various concentrations of MAE (1, 10, or 100 μg/mL) followed by stimulation with LPS (1 μg/mL) for 24 h. LPS strongly stimulated nitrite production (51.01 ± 2.178 nM) in RAW 264.7 cells. By contrast, MAE significantly reduced nitrite production (24.20 ± 1.818 nM, *P* < 0.01) at a dose of 100 μg/mL compared with that in the LPS-stimulated RAW 264.7 cells (Figure 2A). LPS-stimulated RAW 264.7 cells had higher PGE_2_ production (9.90 ± 0.957 ng/mL, *P* < 0.05) compared with the untreated control (5.43 ± 0.502 ng/mL), whereas MAE reduced the PGE_2_ level significantly (6.28 ± 0.102 nM, *P* < 0.05) at 100 μg/mL compared with LPS stimulation (Figure 2B).

### MAE inhibits TARC production in TNF-α and IFN-γ stimulated HaCaT cells

**Figure 3:**
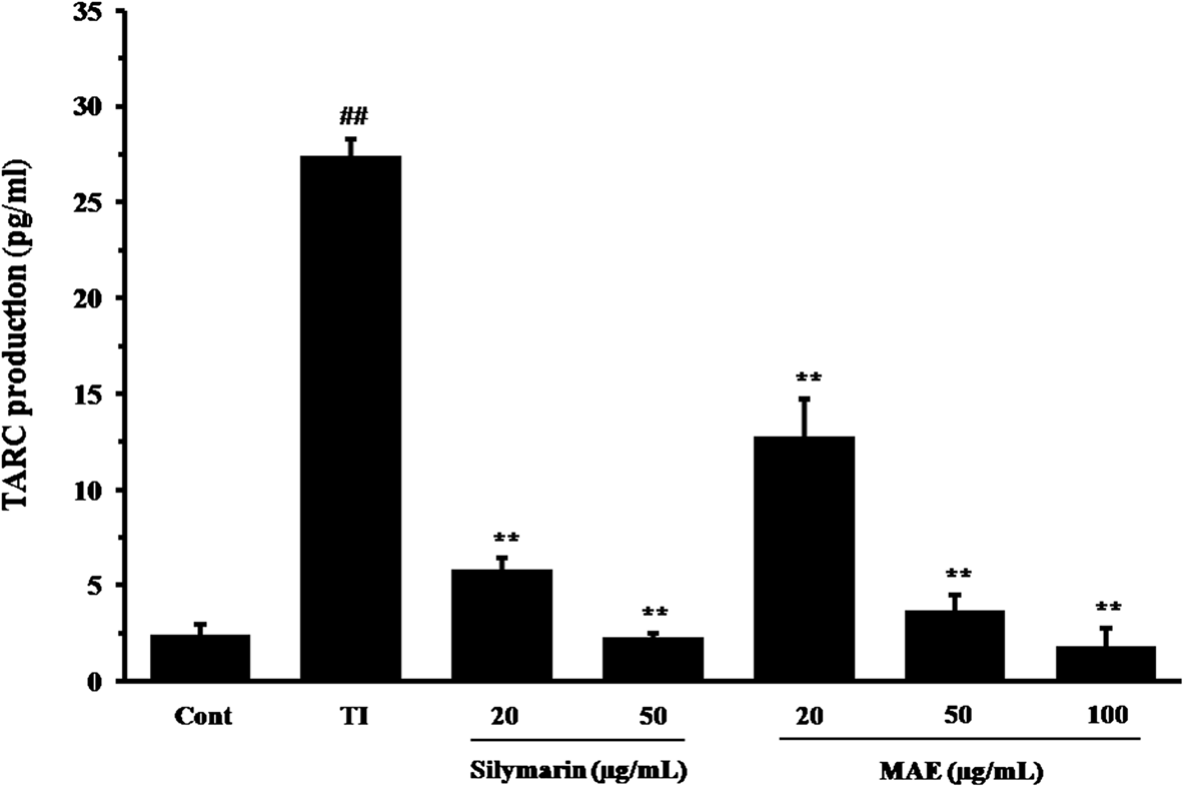
**Effects of MAE on TNF-α- and IFN-γ-stimulated TARC production in HaCaT cells.** The production of TARC was measured using the culture supernatant from the cells co-treated with MAE (20, 50, or 100 μg/mL), and TNF-α and IFN-γ (each 10 ng/mL, TI) for 24 h. Silymarin (20 or 50 μg/mL) was used as a positive control drug. The bar groups represent the means from three independent experiments. ^##^*P* < 0.01 vs vehicle control group; ^**^P < 0.01 vs. TI-treated cells.

We tested the effects of MAE on TARC production in TNF-α and IFN-γ (TI)-stimulated HaCaT cells. The cells were treated with various concentrations of MAE extract (20, 50, or 100 μg/mL) followed by stimulation with TI (10 ng/mL) for 24 h. MAE suppressed TI-stimulated TARC production in a dose-dependent manner (Figure 3). The TI-treated cells exhibited a significant increase in TARC production (24.43 ± 0.879 pg/mL, *P* < 0.01) compared with the untreated control cells (2.44 ± 0.437 pg/mL). The increased of TARC production was reduced to 12.79 ± 1.955 pg/mL, 3.69 ± 0.835 pg/mL, and 1.83 ± 0.895 pg/mL by MAE treatment (*P* < 0.01) at 20, 50, or 100 μg/mL, respectively.

### MAE suppresses the severity of dermatitis and skin lesions in Biostir-AD-induced atopic dermatitis using NC/Nga mice

**Figure 4:**
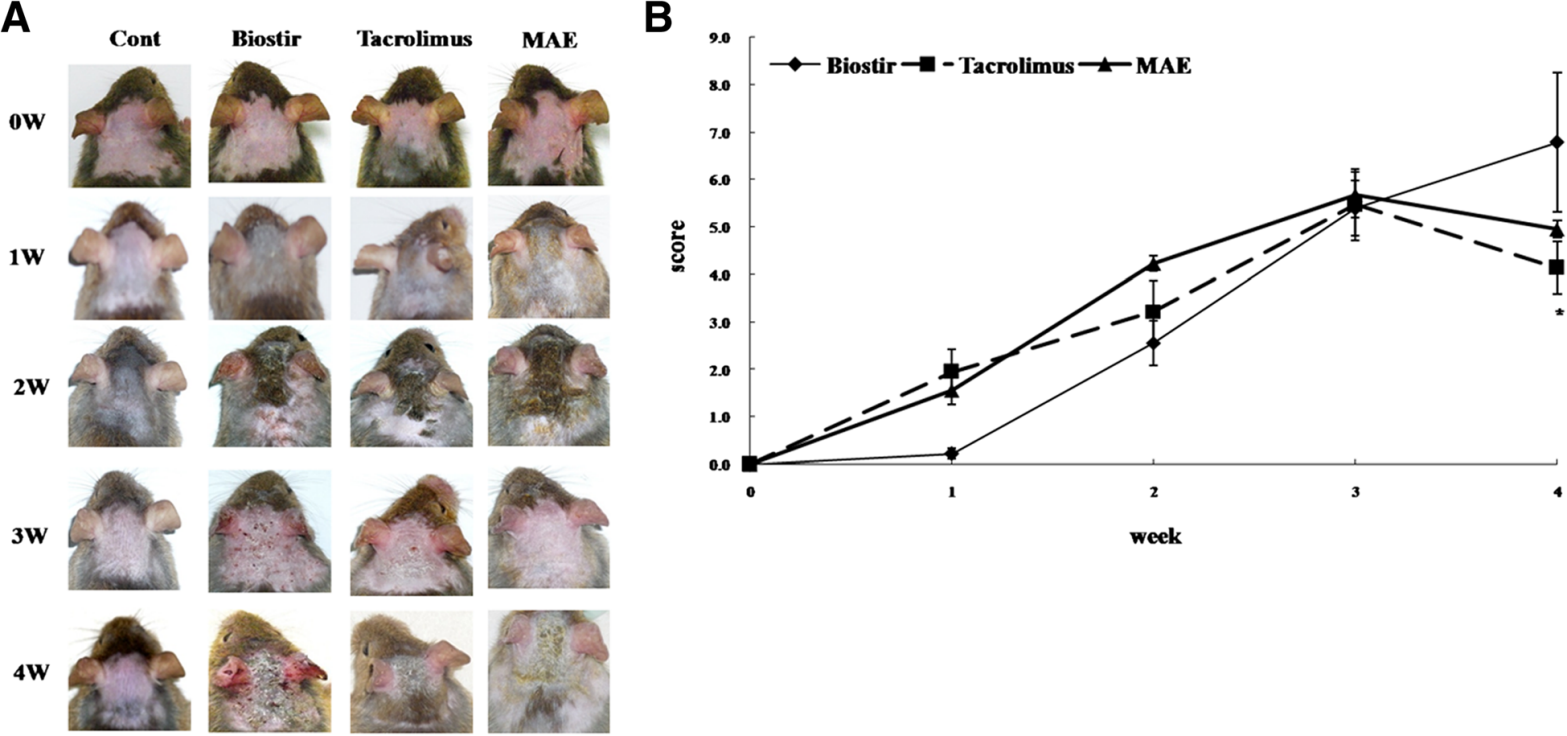
**Effects of MAE on the development of atopic dermatitis, which was induced using a house dust mite extract in NC/Nga mice.** The house dust mite extract was administered to NC/Nga mice to establish an atopic dermatitis animal model. The mice were divided into four groups: normal control group (vehicle-treated mice; 70% ethanol, 200 μL/mouse), Biostir group (Biostir-AD plus vehicle-treated mice), Tacrolimus group (Biostir-AD plus Tacrolimus ointment-treated mice; 50 mg/mouse), and MAE group (Biostir-AD plus MAE-treated mice; 10 mg/mouse). The clinical features **(A)** and dermatitis scores **(B)** were assessed using the criteria described in the Materials and Methods. The features were monitored once each week for four weeks. The values are expressed as the mean ± SEM (n = 5). ^*^P < 0.05 vs. Biostir-AD-treated mice.

The severity of dermatitis was evaluated once each week. The major clinical signs and symptoms developed shortly after the mite antigen was applied to the backs of the mice. The atopic dermatitis‒like skin lesions worsened progressively for four weeks after the initial treatment. Repeated application of Biostir-AD ointment induced skin dryness, followed by mild erythema, hemorrhage, and edema (Figure 4A). Finally, the skin became thick, with severe erythema, hemorrhage, edema, scarring, erosion and excoriation. However, the application of MAE suppressed these skin symptoms. The dermatitis score of each group was determined by summing the eczema area severity index (EASI) values of each mouse (Figure 4B). In the Tacrolimus group, the symptoms increased for three weeks but declined thereafter. In the MAE group, the dermatitis score was lower at three weeks compared with the Biostir group. These results indicate that MAE suppressed the spontaneously induced dermatitis in NC/Nga mice.

### MAE attenuates the atopic dermatitis-like lesions induced by Biostir-AD

**Figure 5:**
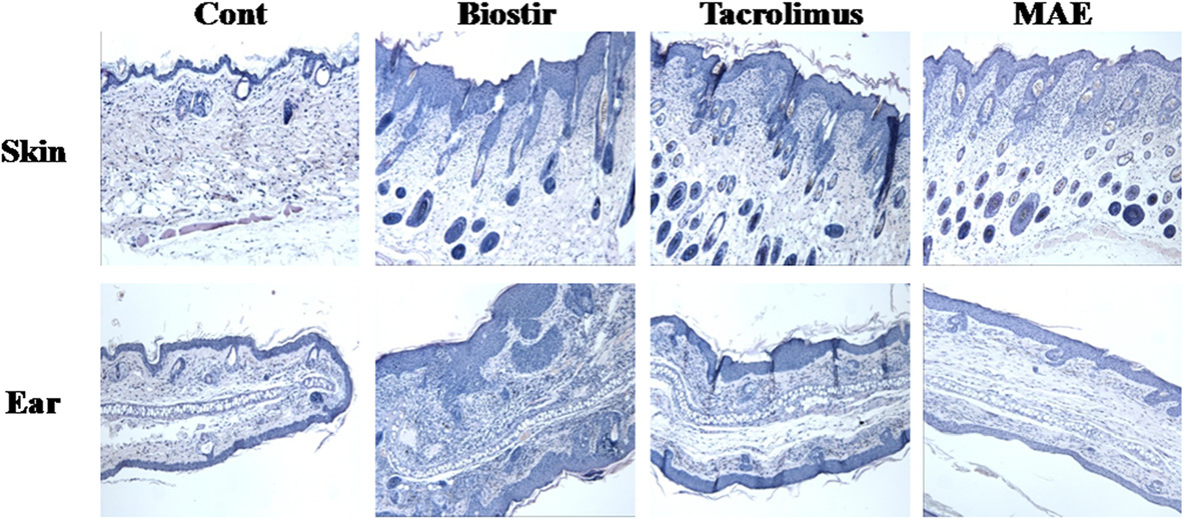
**Effects of MAE on the histological features of house dust mite-induced atopic dermatitis in NC/Nga mice.** The skin and ear tissues were excised from normal control group (vehicle-treated mice; 70% ethanol, 200 μL/mouse), Biostir group (Biostir-AD plus vehicle-treated mice), Tacrolimus group (Biostir-AD plus Tacrolimus ointment-treated mice; 50 mg/mouse), and MAE group (Biostir-AD plus MAE-treated mice; 10 mg/mouse), fixed with 10% formaldehyde, embedded in paraffin, and cut into thin sections. The histological features of the skin and ear lesions were determined after staining with hematoxylin and eosin.

The histopathological features were assessed using specimens from the back skin and ears of NC/Nga mice. As shown in Figure 5, epidermal hyperplasia, parakeratosis, hyperkeratosis, dermal edema, and a large numbers or infiltrated of inflammatory cells were observed in the control group. However, sections of the skin from the back and ears of the MAE-treated group exhibited remarkable reductions in epidermal hyperplasia and cellular infiltration into the dermis. These results suggest that MAE suppressed the Biostir-AD-induced inflammation. The normal group showed no signs of inflammation.

### MAE inhibits the IgE and histamine levels in Biostir-AD-induced atopic dermatitis in NC/Nga mice

**Figure 6:**
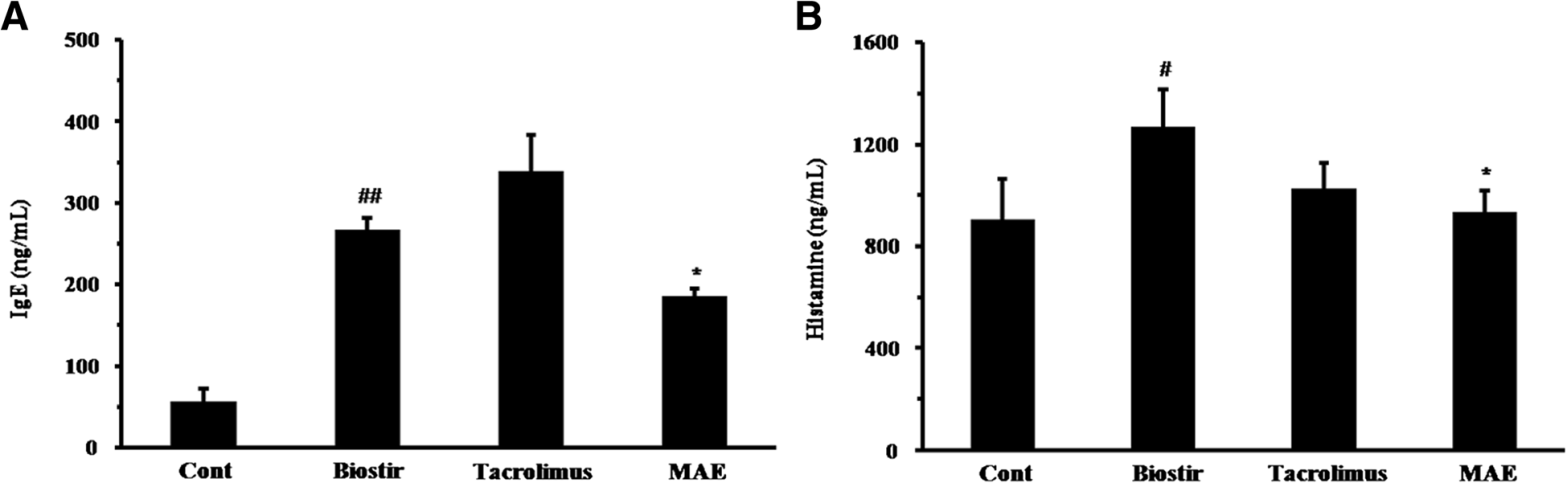
**Effects of MAE on the plasma levels of IgE and histamine in house dust mite-induced atopic dermatitis in NC/Nga mice.** The IgE and histamine levels were measured in plasma samples from normal control group (vehicle-treated mice; 70% ethanol, 200 μL/mouse), Biostir group (Biostir-AD plus vehicle-treated mice), Tacrolimus group (Biostir-AD plus Tacrolimus ointment-treated mice; 50 mg/mouse), and MAE group (Biostir-AD plus MAE-treated mice; 10 mg/mouse). The plasma concentrations of IgE **(A)** and histamine **(B)** were measured by ELISA. The values are expressed the as mean ± SEM (n = 5). ^#, ##^*P* < 0.05 and *P* < 0.01 vs control group, respectively; ^*^P < 0.05 vs Biostir-AD-treated mice.

The plasma levels of total IgE and histamine were determined in the NC/Nga mouse model. We found that Biostir-AD (266.53 ± 14.92 ng/mL, *P* < 0.01) increased the level of IgE compared with the control group (56.67 ± 14.91 ng/mL), whereas the IgE levels in mice administered with MAE (186.01 ± 8.07 ng/mL, *P* < 0.05) were lower than those in the Biostir group (Figure 6A). The MAE-treated group (932.47 ± 84.89 nM) had lower histamine levels compared with the Biostir group (1266.69 ± 147.25 nM) (Figure 6B), although the difference was not significant. These results demonstrate that MAE prevented the development of atopic dermatitis in NC/Nga mice.

## Discussion

Atopic dermatitis is a chronic inflammatory skin disease, which is characterized by pruritic and eczematous skin lesions. Recently, atopic dermatitis has been increasing in industrialized countries, and it is a multifactorial disease with genetic and environmental components, such as infectious agents, food allergens, and aeroallergens. These environmental components elevate the serum IgE levels, which can be a strong trigger of infiltration by T cells, eosinophils, mast cells, macrophages, and keratinocytes [2].

Macrophages have a central role in the production of proinflammatory mediators during the inflammation response. Of the various proinflammatory mediators, nitrite and PGE_2_ have been found to be important in several physiological processes, including vasodilation, neurotransmission, blood coagulation, and immune regulation [19]. NO is synthesized by inducible NO synthase (iNOS), while PGE_2_ is produced by cyclooxygenase 2 (COX-2) [20,21]. They aggravate the inflammatory responses of atopic lesions, which are important feature is of atopic dermatitis [22]. In our study, we found that MAE reduced NO and PGE_2_ production in a dose-dependent manner in LPS stimulated RAW 264.7 macrophage cells.

Keratinocytes produce chemokines and these factors are involved with the development of inflammatory skin diseases, such as atopic dermatitis [23]. Chemokines can lead to a Th1/Th2 imbalance, which results in the development of atopic dermatitis lesions [4]. The lymphocytes that are directed to the skin have recently been shown to express CC chemokine receptor 4, which is the receptor for the CC chemokine TARC. TARC is overexpressed by keratinocytes, especially in the epidermis of atopic dermatitis lesions in the murine model and atopic dermatitis patients [2]. In this study, MAE inhibited TARC production in TI-stimulated HaCaT cells. Overall, these results suggest that MAE is effective in suppressing inflammatory responses and the development of atopic dermatitis by reducing TARC production.

NC/Nga mice have been used as an animal model of atopic dermatitis and share the clinical features of human atopic dermatitis [24]. NC/Nga mice with atopic dermatitis exhibit various histopathological and pathophysiological changes compared with normal mice [25]. The elevation of proinflammatory cytokines, IgE, and histamine are important features in the pathophysiology of NC/Nga mice with atopic dermatitis [4]. Inflammatory cell infiltration, epidermis hypertrophy, intracellular edema, and hyperkeratosis are mainly observed in atopic dermatitis skin lesions [26]. Our study also observed the characteristic pathophysiological alterations of atopic dermatitis. NC/Nga mice where atopic dermatitis was induced by house dust mites exhibited increased levels of IgE and histamine in their plasma compared with normal mice. They also exhibited epidermis hypertrophy, intracellular edema, and infiltration of inflammatory cells in their skin lesions. By contrast, MAE-treated NC/Nga mice had reduced histopathological changes in their skin lesions and lower plasma IgE and histamine levels. Based on these findings, a topical application of MAE may be effective for suppressing the atopic dermatitis induced by house dust mites.

Topical steroids, emollients, and oral anti-histamines are used as the first-line therapy for atopic dermatitis [27]. Steroids are generally prescribed to control the symptoms of atopic dermatitis, but their repeated use can cause severe skin atrophy, susceptibility to infections, and other side effects. These profound side effects mean that chronic usage must be actively avoided. Thus, there is a strong need for the development of a herbal medicine that is effective for the treatment of atopic dermatitis, which should be safe and eco-friendly.

## Conclusions

Our study demonstrated the inhibitory effects of MAE on atopic dermatitis. The inhibition of NO and PGE_2_ production was responsible for the anti-inflammatory effects of MAE, which may be associated with its inhibitory effect on TARC production by keratinocytes in dermatitis lesions. MAE also appeared to reduce the skin symptoms of atopic dermatitis, such as erythema, hemorrhage, and edema. The topical application of MAE significantly reduced the development of house dust mite-induced atopic dermatitis in NC/Nga mice. Overall, these findings provide scientific evidence that MAE has the potential to be developed as a therapeutic agent for the treatment of atopic dermatitis.

## Competing interests

The authors have declared that no competing interests exist.

## Authors’ contributions

HSL and HKS participated in the design of the study data analyses and manuscript preparation. HSL, HH, HL, JKL, and MYL conducted the biological examination of *in vitro* and *in vivo*. All authors read and approved the final manuscript.

## Pre-publication history

The pre-publication history for this paper can be accessed here:

http://www.biomedcentral.com/1472-6882/14/139/prepub
